# Frequent and Unexplained Falls: A Case of Progressive Supranuclear Palsy

**DOI:** 10.7759/cureus.72503

**Published:** 2024-10-27

**Authors:** Cláudia Diogo, Carolina Fernandes, Luís Luz, Sandra Cunha, Joana Raquel Monteiro

**Affiliations:** 1 Internal Medicine Department, Centro Hospitalar de Leiria, Leiria, PRT

**Keywords:** differential diagnosis, good clinical practice, mri- magnetic resonance imaging, parkinson' s disease, progressive dementia, progressive supranuclear palsy (psp)

## Abstract

Progressive supranuclear palsy (PSP) is the most common neurodegenerative form of atypical parkinsonism. Although its prevalence has increased recently, it remains underdiagnosed. PSP is characterized by parkinsonism, downward gaze disorder, and a tendency to fall due to degeneration of the basal ganglia, brain stem, and cerebellum. Various clinical presentations have been linked to this entity, often making its diagnosis difficult, which can take three to four years to be established. PSP diagnosis mainly relies on clinical data and can only be confirmed with the neuropathological findings at autopsy. However, as per recent research, neuroimaging, namely brain MRI, can aid in an earlier diagnosis.

We report the case of an 85-year-old female with a history of recurrent and unexplained falls over the last two years that had been wrongly attributed to Parkinson’s disease (PD). She initially presented complaining of recurring falls associated with retropulsion movements that got progressively worse and eventually was not able to walk on her own. Concomitantly, there was also a cognitive dysfunction with frontal predominance. An exhaustive physical examination and clinical history revealed indicators consistent with PSP.

PSP diagnosis demands a high clinical suspicion, and hence clinicians should have a good understanding of this disease for its early recognition. Although it is a devastating disorder, and no medications that can reverse the disease course are currently available, early diagnosis of PSP may contribute to improving the quality of life of the patients and their families, and prevent complications. This report highlights the clinically significant improvement in motor and neuropsychiatric symptoms when a multidisciplinary medical team is involved in the care of a PSP patient.

## Introduction

Progressive supranuclear palsy (PSP) is the most common neurodegenerative form of atypical parkinsonism, which appears in adulthood, usually after the age of 40 years [1]. Although its prevalence has been on the rise in recent years (7:100,000), it remains an underdiagnosed disease [2]. It is characterized by parkinsonism, vertical supranuclear palsy with downward gaze abnormalities, and a tendency to fall due to postural instability secondary to a degeneration of the basal ganglia, brain stem, and cerebellum. However, besides this classic form, PSP presentation can be heterogeneous [1], which varies throughout the disease's progression, often making its diagnosis difficult. In the early stages, distinguishing parkinsonism from other related disorders is challenging due to the lack of specific clinical findings, and it generally takes three to four years to reach a diagnosis [2,3]. Patients may have a symmetric rigidity or bradykinesia in the axial muscles, especially in the neck and upper chest, apart from cognitive dysfunction with a frontal predominance (language and behavioral changes), early dysphagia, and dysarthria [1-6]. PSP inevitably leads to a decline in the quality of life of patients, with the median survival time ranging between five and eight years from the onset of the illness [7].

PSP diagnosis mainly relies on clinical data and can only be confirmed based on neuropathological findings at autopsy [5,8]. No laboratory or imaging studies can establish the diagnosis. However, according to recent research, neuroimaging, namely brain MRI, helps with an earlier diagnosis by combining neuropathological and clinical data [5]. Brain MRI can identify mesencephalic atrophy that is characteristic of PSP and is recognized as hummingbird sign and morning glory sign [5,6]. These findings considerably improve the accuracy [6] in the differential diagnosis of the various parkinsonism disorders with clinical manifestations similar to PSP [1,3,5-6,9], such as Parkinson’s disease (PD) [9]. While these MRI findings are also seen in vascular parkinsonism, idiopathic normal pressure hydrocephalus (iNPH), and dementia with Lewy bodies (DLB) [9-10], distinguishing them remains a challenge. However, with the development of manual and automatic volumetric brainstem measurement tools, MRI can distinguish among the various dementia syndromes such as PSP, PD, and DLB [9-10], facilitating and improving diagnostic accuracy.

Despite the recent scientific advances, PSP continues to be a challenging diagnosis that demands high suspicion. This report discusses a case of PSP in an 85-year-old female with a history of recurrent falls.

## Case presentation

The patient was an 85-year-old woman, a retired teacher, with a history of recurrent and unexplained falls over the last two years that were wrongly attributed to PD. She initially presented with recurring falls associated with retropulsion movements that got progressively worse and she was eventually not able to walk on her own. There was no history of tremors, autonomic dysfunction, hallucinations, palpitation, or syncope.

The patient had been diagnosed with PD a year before and was treated with levodopa/carbidopa, without any improvement. There was a cognitive decline with memory loss and reading difficulty, as well as impairment in performing activities of daily living such as the inability to eat alone (“dirty tie sign” positive while eating). In the last two years, she had experienced several falls with consequent head injuries and rib fractures. These falls were not associated with amnesia. On physical examination, there were no visual acuity or visual field defects. Still, she presented with a vertical supranuclear gaze palsy and a decrease in the speed of vertical saccadic eye movements. She was unable to look up or pick up an object from the floor; she couldn’t flex her cervical spine (representing resistance to passive neck movement) and had a tendency to fall backward; facial expression was reduced, which indicated dystonia. Increased rigidity of the chest was noticed, especially when standing up.

**Figure 1:**
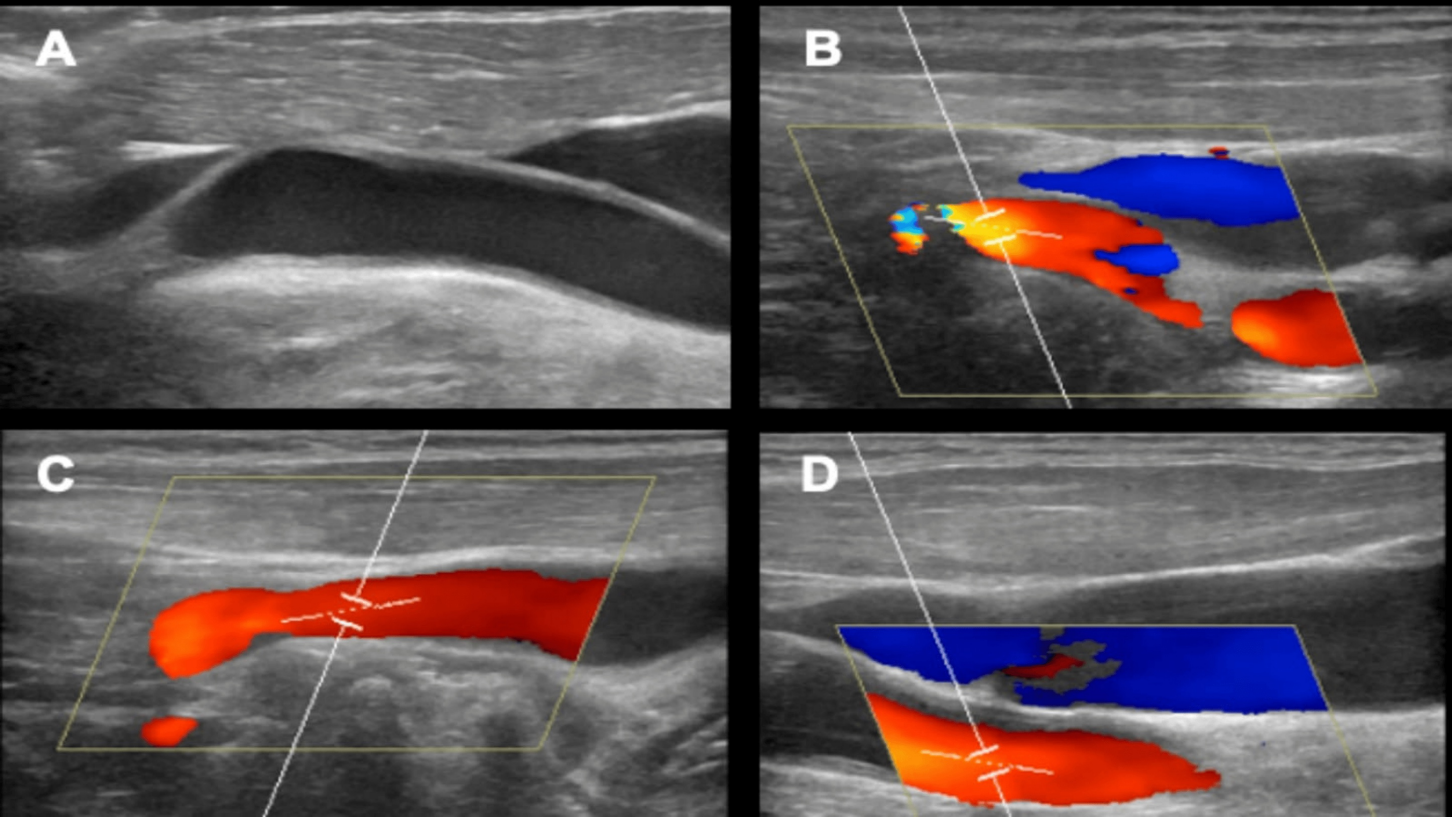
Carotid artery and vertebral ultrasound image of the patient Morphological study: no anatomical changes relevant (A: carotid morphological study). Doppler study: no stenosis with hemodynamic significance. The vertebrae are permeable and have a cephalic flow direction (B: left internal carotid artery; C: right common carotid artery; D: left common carotid artery)

The patient underwent a comprehensive blood analysis (Table 1) and carotid Doppler ultrasound, which were within normal limits (Figure 1).

**Table 1:** Results of extensive blood tests performed for differential diagnosis ALT: alanine transaminase; ANA: antinuclear antibody test; AST: aspartate transferase; C3: complement component 3; C4: complement component 3; CRP: C-reactive protein; CSF: cerebrospinal fluid; ESR: erythrocyte sedimentation rate; GGTP: gamma-glutamyl transpeptidase; HbA1C: glycated hemoglobin; HIV: human immunodeficiency virus; RF: rheumatoid factor; TSH: thyroid-stimulating hormone

Test	Results	Reference range
White blood cells	6.1 x10^3^/μL	4.0-10.0
Neutrophils	4.5 x10^3^/μL	1.8-8.0
Lymphocytes	1.7 x10^3^/μL	1.5-6.5
Monocytes	0.7 x10^3^/μL	0.0-0.8
Hemoglobin	12 g/dL	11.5-16.0
Platelets	220 x10^3^/μL	150-500
Glucose	5.5 mmol/L	4.1-5.9
Creatinine	0.85 mg/dL	0.51-0.95
Urea	7.0 mmol/L	2.8-7.2
Sodium	137 mmol/L	136-146
Potassium	4.6 mmol/L	3.5-5.1
Magnesium	0.83 mmol/L	0.73-1.06
Ionized calcium	1.16 mmol/L	1.15-1.30
Albumin	36 g/L	35-52
Bilirubin	11.1 mmol/L	5.0-21.0
ALT	5 U/L	3-34
AST	16 U/L	15-35
GGTP	12 U/L	<38
ANA	Negative	Positive ≥160
RF	Ul/mL	<14
C3	134 mg/dL	90-180
C4	30 mg/dL	10-40
CRP	3.5 mg/L	<5.0
ESR	7 mm	<10
Protein electrophoresis	Normal	
TSH	2.75 μUI/mL	0.38-5.33
Vitamin B12	257 ng/mL	180-914
Folate	6.7 ng/mL	>2.3
Hepatitis B virus	Negative	
Hepatitis C virus	Negative	
Syphilis serology	Negative (<0.1)	<0.9 negative
HIV serology	Negative	
HbA1C	7.3%	
CSF	Límpido	
Leukocytes – manual	0/mm^3^	
Non-leukocyte cells	0	
Erythrocytes	0	
Glucose	65 mg/dL	40-70 mg/dL
Total proteins	300.0 mg/dL	

**Figure 2:**
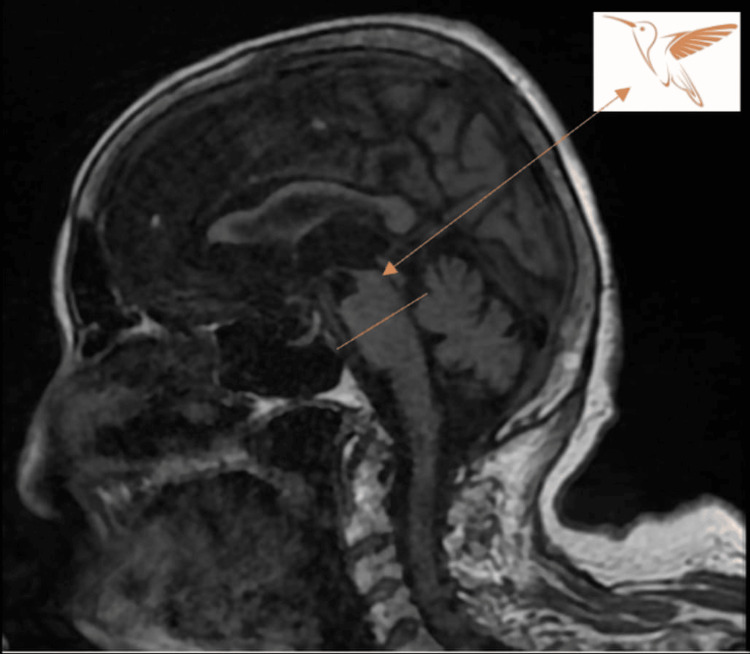
Hummingbird sign The sagittal T1 MRI image shows selective pronounced atrophy of the midbrain, with mesencephalic predominance and preservation of pons, which is concave at its cranial margin - the characteristic hummingbird sign in the PSP MRI: magnetic resonance imaging; PSP: progressive supranuclear palsy

**Figure 3:**
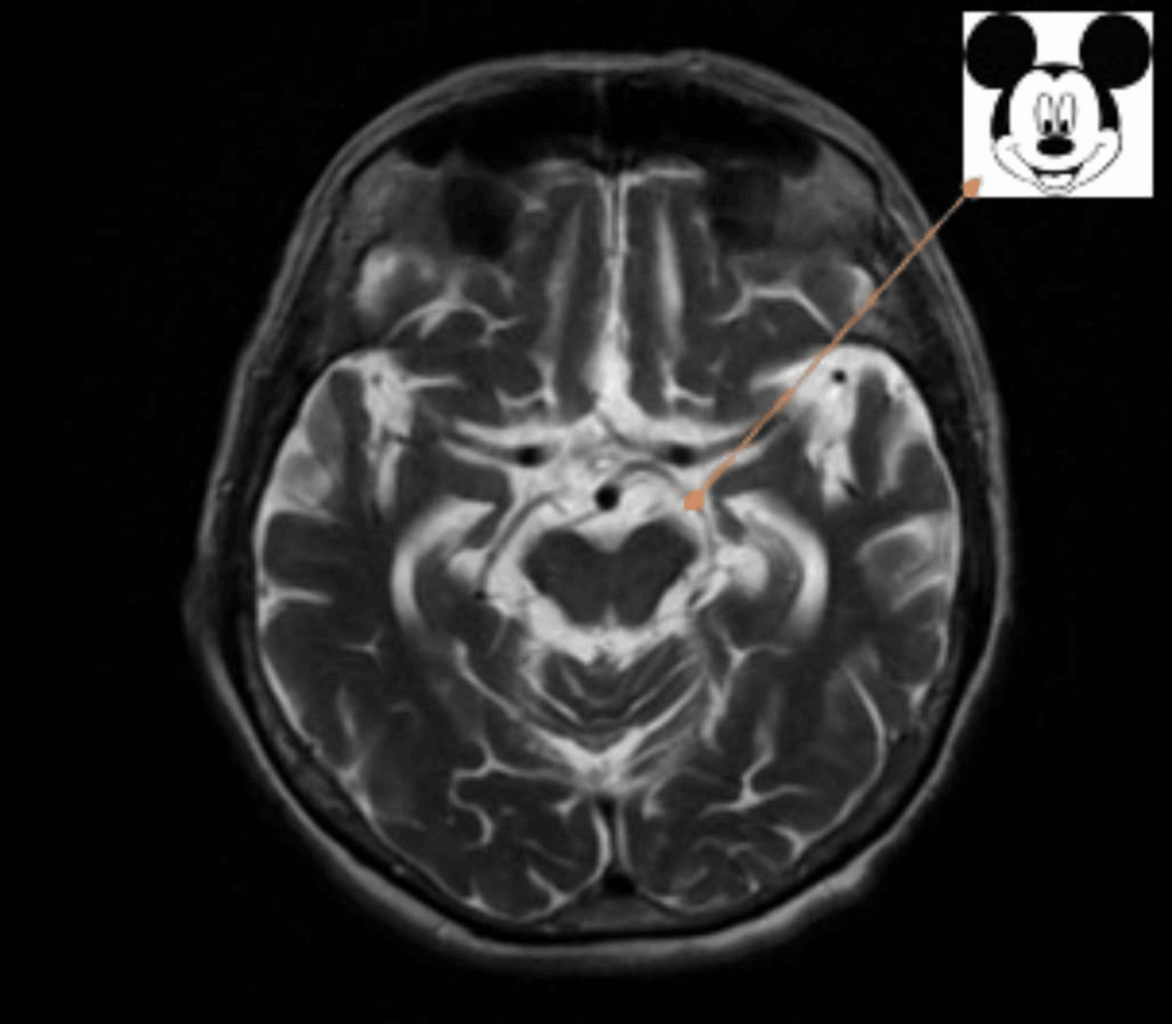
Morning glory and Mickey Mouse sign The axial T2 MRI image shows a reduction in the anterior-posterior diameter of the midbrain (atrophy of the midbrain tegmentum) with thinning of cerebral peduncles giving the appearance of the morning glory sign and the Mickey Mouse sign, again characteristic of PSP MRI: magnetic resonance imaging; PSP: progressive supranuclear palsy

The patient also underwent an MRI brain, which revealed several abnormal findings: in the sagittal view, there was pronounced brainstem atrophy, with mesencephalic predominance and relatively preserved pons, giving an appearance of the head and body - the characteristic hummingbird sign in the PSP (Figure 2); in the axial view, there was a reduction in the anterior-posterior diameter of the midbrain with thinning of cerebral peduncle giving the appearance of morning glory sign and Mickey Mouse sign (Figure 3).

**Figure 4:**
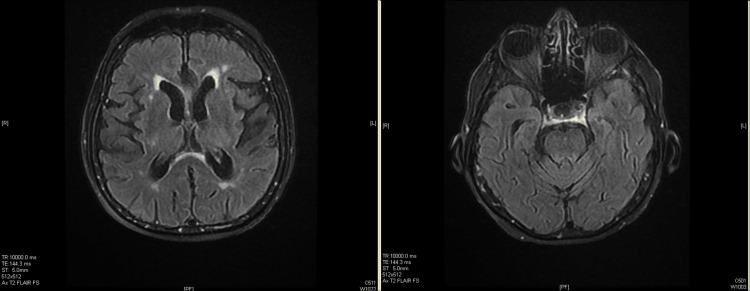
Brain magnetic resonance angiography (MRA) Brain magnetic resonance angiography (MRA) was unremarkable

Brain magnetic resonance angiography (MRA) was unremarkable (Figure 4). An MRI brain showed mild periventricular leukomalacia on the T2 FLAIR sequence. 

We ruled out environmental factors, medications, infection of the central nervous system, and other neurodegenerative diseases such as Parkinson's disease, corticobasal degeneration, spinocerebellar degeneration, and Lewy body dementia. Based on the clinical presentation, neurological examination, and MRI brain findings, the diagnosis of PSP was established.

## Discussion

We report a case of PSP in an 85-year-old female with gait disturbances and postural instability leading to recurrent falls, symmetrical axial rigidity (not appendicular asymmetric rigidity like in PD) [4,5], and dysphagia. The patient had a poor response to carbidopa/levodopa, which also excluded PD [1-5].

PSP is an underdiagnosed disease that demands clinical suspicion. One of the most common and classical clinical presentations of PSP is recurrent falls due to a decreased perception of danger associated with reduced frontal lobe function. Therefore, PSP falls are more likely to result in severe trauma when compared to other neurodegenerative diseases [3,4,11]. Like in this clinical case and according to Kaneko et al., despite several prior episodes of falls, severe trauma was needed for PSP diagnosis to be considered [12]. We believe that the delay in diagnosis was due to the clinicians' lack of awareness of this entity and their difficulty in differential diagnosis with other dementia syndromes such as idiopathic Parkinson's disease (IPD), multi-systemic atrophy, Alzheimer's disease, dementia with Lewy bodies (DLB), and corticobasal or vascular parkinsonism [1,3,5,12]. The patient was misdiagnosed with PD, with an incomplete study of the recurrent falls describing that they were assumed to be related to IPD.

PSP diagnosis demands the combination of a thorough medical history with a detailed neurologic examination combined with diagnostic tools such as brain MRI [1-12]. The characteristic sign that can be found on the brain MRI is the hummingbird sign, which results from midbrain tegmentum pronounced atrophy detected in the sagittal view [3,9,10], unlike moderate brainstem atrophy that can be found in DLB patients and non-significant atrophy seen in PD patients [10]. However, the identification of this imaging feature relies on visual judgment, and currently, there are no defined criteria for its determination. According to Müller et al., the measurement of diameter, volume, and area ratio of midbrain/pons can help differentiate between PD and PSP; however, it requires an experienced neuroradiologist [10].

## Conclusions

PSP is a neurodegenerative condition that is frequently misdiagnosed. Its diagnosis demands a high clinical awareness, and hence a thorough knowledge of this disease is essential for its early recognition. Although it is a devastating disorder, and no medications can reverse the disease course, early diagnosis of PSP may contribute to improving the quality of life of the patients and their families, and prevent possible life-threatening complications. This report highlights the clinically significant improvement in motor and neuropsychiatric symptoms when a multidisciplinary medical team treats a PSP patient. The multifaceted approach, with the participation of a multidisciplinary medical team, made a huge difference in the patient's life.
